# The extent and predictors of discrepancy between provider and recipient reports of informal caregiving

**DOI:** 10.1016/j.socscimed.2021.113890

**Published:** 2021-04-02

**Authors:** Sean Urwin, Yiu-Shing Lau, Gunn Grande, Matt Sutton

**Affiliations:** a Health Organisation, Policy and Economics, School of Health Sciences, University of Manchester, Manchester, UK; b Division of Nursing, Midwifery and Social Work, Manchester Academic Health Science Centre, University of Manchester, Manchester, UK; c Melbourne Institute, Applied Economic and Social Research, University of Melbourne, Melbourne, Australia

**Keywords:** Informal care, UKHLS, Measurement, Ageing, Long-term care

## Abstract

Informal care research mainly relies upon carers reporting that they provide this type of care. Little is known about whether reports from recipients would produce similar information. We explore whether providers and recipients are in agreement with each other’s reports of informal care at the extensive and intensive margin and whether particular characteristics of providers and recipients predict any discrepancies. Using data from the 2015–2017 wave of the UK Household Longitudinal Study (UKHLS), we find that among those who reported receiving informal care a provider confirmed only 37.5% of these. Each additional restriction on activities and instrumental activities of daily living for a recipient increases the probability of agreement by 5.2 and 9.3 percentage points, respectively. When both parties report informal care, providers report on average 10.55 (37%) more hours per week compared to recipients. This represents an annual difference of £12,081 using the replacement monetary valuation method. If we rely on recipient reports, we may be more likely to capture how many in the population are caregivers. However, we may also be less likely to capture the full hours of care for each caregiver. These discrepancies in reported caregiving affect studies of the consequences of caregiving and economic evaluations of interventions that impact on caregiving.

## 1 Introduction

Research on non-market activities relies on respondents to indicate that they have performed these types of activities. Informal care is a non-market activity and information regarding this type of care is often measured through surveys. We define this type of care, which can also be referred to as unpaid care, as “people providing any help to older family members, friends and people in their social network, living inside or outside of their household, who require help with everyday tasks.” (OECD, 2019). Correct identification of who is providing or receiving informal care is paramount if carer policy, care planning and support for informal carers are to be robustly evaluated through surveys.

Given that informal care involves more than one person (a provider and a recipient) this offers two possible sources for collecting information. Does measuring informal care from two perspectives result in two different accounts? One problem with informal care information, compared to income information, for example, is the lack of a feasible means of verifying provider or recipient reports to determine the ‘truth’. Suitable administrative data on carer and recipient benefit allowances requires perfect uptake for use as a comparator. Often this is not the case among countries that provide these benefits (Courtin et al., 2014). Alternative administrative data is reliant on contact with formal care providers (Lemmon, 2020).

One method to overcome this verification issue would be to observe caregiving taking place with a pre-formulated definition of caregiving. This would likely be intrusive for many, costly to implement (Juster and Stafford, 1991) and would suffer from small samples as found in Wimo et al. (2010) and Wimo and Nordberg (2007). It may be more feasible to use the idea of multiple observers which would exploit the multiple observer/participant nature of caregiving as both providers and recipients are involved in this non-market exchange. Taking this approach could help identify additional caregiving that otherwise would not have been recorded by using only one perspective.

Both providers and recipients may not identify as giving or receiving care for a variety of reasons. Providers may not wish to identify as a ‘carer’ (Carduff et al., 2014; Corden and Hirst, 2011) or some may struggle to see emotional support as legitimate caregiving (Knowles et al., 2016). Providers may also not recognise that they perform caregiving duties as they may struggle to differentiate between ‘normal’ and ‘caregiving’ related activities (van den Berg and Spauwen, 2006). Recipients, however, might not identify as receiving care due to a general desire to retain a sense of independence (Grande et al., 1997). Further, the complexity of intra-family roles and caregiving responsibilities (Lingler et al., 2008) as well as the previously mentioned issues may be contributing factors as to why a recipient nominates someone in their social network as their carer but this person does not confirm this claim and vice versa.

The largest source of informal care information at the population level is from censuses. Censuses that include a question on informal care among English speaking countries ask an individual to state if they are a *provider* of informal care but do not ask about *receipt* (see United Nations, 2019). They often find that a substantial proportion of the population identify as providing informal care. For example, there were approximately 5.8 million carers (10.3% of the population) in England and Wales in 2011 (ONS, 2013), 2.1 million (11.3% of the population) in 2016 in Australia (ABS, 2016), 0.4 million (12.1% of the population) in 2013 in New Zealand (NZ Carers Alliance, 2014) and 0.2 million carers (4.1% of the population) in 2013 in the Republic of Ireland (Central Statistics Office, 2016). Therefore, any discrepancy in caregiving reports between providers and recipients will apply to a large number of people throughout the world. Evidence based on survey data suggests that caregiving in the UK may have increased to 7.6 million in 2015/17 from 6.5 million in the 2011 census (Social Market Foundation, 2018).

The majority of studies which have explored the effect of informal care provision on the health, wellbeing and labour market outcomes of the provider have used caregiving information from the provider as these were the subject of analysis (Coe and Van Houtven, 2009; Heitmueller, 2007; Heitmueller and Inglis, 2007; Leigh, 2010; Schmitz and Westphal, 2017; van den Berg et al., 2014). Reports from recipients have typically been used when analysing the effect of informal care on the demand for formal care and health care (Bolin et al., 2008; Bonsang, 2009; Van Houtven and Norton, 2004). Mommaerts and Truskinovsky (2020) is an exception which used both care provision and receipt (from different datasets) when analysing the effect of the business cycle on informal care.

Substantial discrepancy between provider and recipient reports of informal care could present an important problem for research in this area. If this discrepancy is unrelated to any characteristic of the provider or recipient (nominated or confirmed), then the consequence of this for empirical analysis would be added noise. Alternatively, if the discrepancy is related to a particular characteristic then the effect of informal care would result in a biased sample with over or under-representation of certain types of caregivers or recipients. Misclassified carers may produce a biased result when attempting to identify the causal effect of providing informal care on other outcomes, such as work, health and health care. For example, if a sub-group of carers are misclassified as non-carers, but have better (worse) health than the rest of the sample, then the negative health effects of caregiving usually found in the literature may be overstated (understated).

Only one study has compared reports from the provider and recipient. Rutherford and Bu (2018) explored the extent and characteristics of discrepancy in informal care provision and receipt among spousal dyads aged 50 years old and over using the English Longitudinal Study of Ageing (ELSA). Rutherford and Bu (2018) found that among claims of care made by a recipient, 52.6% had a spousal provider confirm this whereas among provider claims of care the spousal recipient confirmed 81.6% of these. Therefore, provider claims were more likely to be confirmed than recipient claims. Those who received a large number of hours as reported by the recipient, females and recipients in worse health were more likely to have their claim confirmed. They also found that carers tended to report more hours than their spousal recipient did.

In this paper, we investigate if reports of informal care supply at the extensive (confirmed or unconfirmed reports of care) and intensive (reported hours of care) margins differ according to the accounts of the provider or recipient who co-reside with one another. At the extensive margin, we explore the degree of discrepancy and predictors which are associated with it. This extends the work of Rutherford and Bu (2018) in several ways. First, through the use a larger sample of respondents in the UK Household Longitudinal Study (UKHLS) that is not restricted to analysis between spouses as in ELSA. In terms of the methods, we categorise dyads into three types: those unconfirmed by the provider, those unconfirmed by the recipient and those confirmed by both provider and recipient. This enables the identification of carer or recipient sub-groups across three dyad types that may not be identified from one perspective. We analyse these dyads types jointly using a multinomial logistic regression which includes predictors from both the provider and recipient side. These include additional variables to Rutherford and Bu (2018) such as the dyad relationship type, region, household size, benefit receipt and characteristics of the interview. We further extrapolate our extensive margin results of discrepancy to a national level to quantify the number of carers that may not be identified if only provider or only recipient information is used. This is relevant to understanding the totality of informal care taking place in society.

At the intensive margin, we explore discrepancy in reported hours of informal care. We extend the literature in this area by examining the difference in hours within a care dyad and the predictors of hour’s discrepancy. We demonstrate the implications of this discrepancy with a replacement cost calculation. Analysis at the intensive margin has important implications for those using time costs, particularly for economic evaluations.

## 2 Data

We use the UKHLS which began in 2009 (University Of Essex, 2019). It is primarily carried out using computer assisted face-to-face interviews with trained interviewers, although in some cases this is done over the telephone or via a web interview. An important feature of relevance to this study is that on first contact interviewers obtain basic information on all members of a household, such as age and gender, from one of the household members. On subsequent visits to the household, a more extensive interview is carried out with all consenting household members. A further feature of the survey’s data collection is the proxy interview. This separate, more limited questionnaire is given to a proxy respondent in place of the intended respondent.

We use wave seven (2015–2017) of the UKHLS as this includes questions on both the provision and receipt of informal care. An advantage of the UKHLS is its large panel that contains roughly 28,000 households and depth of information available for analysis. This makes it best placed to address the current research question compared to other surveys such as ELSA, Health Survey England and the Family Resources Survey because it strikes a better balance between a large sample of respondents whilst retaining sufficiently detailed information. Given that only members of the household are interviewed, we can only compare reports of provision and receipt where both the provider and recipient co-reside.

### 2.1 Informal care provision

The UKHLS asks provision questions in all of its waves. These provision questions come before any receipt of informal care questions. Respondents are asked:

“Is there anyone living with you who is sick, disabled or elderly whom you look after or give special help to (for example, a sick, disabled or elderly relative, husband, wife or friend etc)?”

It is possible to identify up to sixteen co-residing recipients. If a respondent indicates they are a carer they can record the total hours given to all recipients (both co-residing and non co-residing) in a typical week:

“Now thinking about everyone who you look after or provide help for, both those living with you and not living with you - in total, how many hours do you spend each week looking after or helping them?”

In the proxy questionnaire, the question on the number of hours of care provided is not asked. These proxy respondents are only asked to identify who their respective proxy provides care to within the household.

### 2.2 Informal care receipt

Receipt of informal care is currently available in wave seven of the UKHLS as part of the social care module (University Of Essex, 2019). It is asked along with detailed information on formal home care receipt and payment.

For a respondent to indicate that they receive informal care they first must be at least 65 years old and answer that they require assistance with at least one activity from a list of four instrumental activities of daily living (IADL) and eleven activities of daily living tasks (ADL):

“In the last month who has helped you with personal things around the home including ... ?”

Respondents can then indicate their relationship to the nominated provider along with the person identifier (of co-residing providers) of each provider of activities of daily living and instrumental activities of daily living tasks separately. Hours are reported per provider in the past week:

“Thinking about ..., in the last week, how many hours have they helped you in person with these kinds of tasks?”

### 2.3 Predictors

We include predictors of care receipt and provision based on a review of the literature of relevant studies both in the context of informal care (such as Rutherford and Bu (2018)) and of general inconsistency in survey responses related to health (such as Black et al. (2017) and Jognston et al. (2009)). Some predictors found in the literature are not included in the present study as there was insufficient variation. For example, almost all recipients are retired; therefore including recipient labour force status is unlikely to have any predictive power.

We use two sets of predictors; a basic and an extended set, details of each variable are shown in Table A1. Each set of predictors contains information at an individual (provider or recipient), dyad or household level. The basic predictor set includes information that is available from the initial contact with the household: gender, age (both at provider and recipient level), dyad relationship type (at dyad level), household size and region (both at household level). The extended set includes from both the provider and recipient: ethnic group, degree qualification, whether others were present during the interview and if the respondent had not been interviewed before in the survey. From only the recipient this set includes whether the respondent has memory difficulties, sight difficulties and if they receive attendance allowance. From only the provider side the set includes whether they receive carers allowance, have a health condition and whether they are retired. We further include in this set: household monthly equivalised income (at household level), interview date difference (at dyad level) and the number of interviewer calls to the household (at household level).

## 3 Methods

### 3.1 Classification of informal care dyads

Table 1 presents a framework that groups informal care dyads based on the responses of providers and recipients. A confirmed dyad is where the provider and recipient declare their provision and receipt of care to each other (denoted with a tick). Unconfirmed by recipient dyads are cases where the provider declares they are a caregiver but the nominated recipient does not confirm this claim of provision (denoted with a cross). Unconfirmed by provider dyads are the opposite of unconfirmed recipient dyads where receipt claims are unconfirmed by the nominated provider. No claim dyads are all combinations of individuals where both individuals do not make any claim of provision or receipt.

Importantly, an unconfirmed claim by a member of a dyad may happen for four reasons. The respondent either did not take part in the survey (unit non-response), the survey was completed by a proxy where the relevant questions are not included (proxy non-response), they did not answer the relevant questions (item non-response) or they indicated they did not give or receive care through the relevant questions. We can include unconfirmed members of a dyad if they have completed the basic set of covariates and if a proxy has completed the proxy questionnaire.

### 3.2 Agreement model

Our empirical strategy is to analyse what factors influence whether or not provider and recipient care claims match both at the extensive and intensive margins. We derive a conceptual model for outlining the characteristics of agreement in the identification of informal care from providers and recipients in equation (1):

(1) $$A_{d}=f(X_{r},X_{p},H_{r\;},H_{p},I_{r},I_{p},HH_{d})$$

*A_d_* is an indicator for dyad *d* of whether both the provider and recipient agree with claims of provision and receipt of informal care. This indicator is only calculated for dyads where at least one member of the dyad reports informal care and all members of the dyad are responders. The agreement indicator is a function of socio-demographic characteristics *X*, health *H* and interview circumstances *I* of both the recipient *r* and provider *p* within dyad *d*. Characteristics of the household *HH* are measured only for the dyad as both recipient and provider co-reside in the same household.

### 3.3 Extensive margin analysis

At the extensive margin, we first examine the proportion of the dyads in our sample which fall under the three types: unconfirmed by recipient, unconfirmed by provider and confirmed dyad. This analysis is restricted to care nominations for someone who co-resides in the same household. We calculate the under-reported proportion for providers as $\left(\frac{\sum_{d}P}{\sum_{d}P+\sum_{d}PR}\right)$ by using the total number of unconfirmed by provider dyads $\left(\sum_{d}P\right)$ as a percentage of confirmed $\left(\sum_{d}PR\right)$ and unconfirmed by provider dyad types. The calculation of the under-reported proportion for recipients replaces the summation of unconfirmed by provider dyads with the summation of unconfirmed by recipient dyad types.

Second, to understand the scale of discrepancy we obtain a national estimate of the number of caregiving claims in the UK using equation (2):

(2) $$\theta *r*\frac{\sum_{d}PR+\sum_{d}R+\sum_{d}P}{\sum_{d}PR+\sum_{d}R}$$

We first use the UK 2011 census national figure for the number of carers (denoted *θ*) (Carers UK, 2019) multiplied by the proportion of co-residential carers in the UKHLS (denoted γ) and further multiplied by the number of all claims as a proportion of all provider claims $\left(\sum_{d}PR+\sum_{d}R\right)$. Using the in-sample proportions of each dyad type, we can estimate the national number of unconfirmed and confirmed claims in the UK from the figure derived in equation (2).

Third, we examine the predictors of discrepancy using dyad type as the outcome variable. As there is no implied ranking for each mutually exclusive dyad type a multinomial logit specification is estimated using maximum likelihood (Wooldridge, 2010). The outcome variable takes a value of one if the dyad is unconfirmed by the recipient, two if the dyad is unconfirmed by the provider and three for a confirmed dyad. We report the average marginal effects for each predictor. A positive coefficient for a binary variable for the confirmed dyads outcome means an individual who is part of the binary group (equal to one) has a higher probability of being a confirmed dyad than an individual not part of the binary group.

First, we use the basic set of predictors for analysis, we then conduct analysis on the extended set of predictors which helps to understand whether any of the results from the basic set are due to the omission of other characteristics. We include instrumental activities of daily living and activities of daily living restrictions in two forms across separate regressions. First as counts and second as binary indicators for each separate restriction as there may be particular activities of daily living restrictions that are associated with a dyad type. As a sensitivity check, we apply cross-sectional survey weights on the extended set of predictor results.

We perform two specification tests. The first examines whether it is appropriate to combine two dyad outcome categories together and therefore estimate a single binary model. This is known as the Wald test for combining outcomes (Long and Freese, 2001). We are mainly interested in whether the test provides evidence in favour of having one unconfirmed dyad type rather than the two we estimate. The second tests the independence of irrelevant alternatives assumption. Under this assumption, exclusion of one dyad type should not affect the predictors from the remaining dyad types. We report both the Hausman and McFadden (1984) and Small and Hsiao (1985) tests. The null hypothesis of both these tests indicates that the independence of irrelevant alternatives assumption holds and therefore that the multinomial logit is appropriate. However, the usefulness of these types of tests in applied work has been debated (Cheng and Long, 2016) and as a result we interpret the results from these tests with caution.

### 3.4 Intensive margin analysis

Analysis at the intensive margin explores differences in providers and recipients reported hours (and subsequent monetary valuations) as well as the characteristics associated with large differences. There is partial censoring of the hours of informal care (*y_i_*) for both provider and recipient reports within lower (*m_i_*) and upper bounds (*M_i_*), for individual i:

$$m_{i}\leq y_{i}\leq M_{i}for\;i=1,\;..,I$$

There are two main methods to assign a point value with banded data. The first is to assign a mid-point value to each band. An alternative, used in this study, is to use interval regression to obtain a point value for the hours. Table A2 provides details of the lower and upper values assigned to each band for interval regression. An advantage with the interval regression method over assigning a midpoint value is that information of the provider and recipient can be used to generate the prediction. This regression method assumes the error term is normally distributed and is estimated via maximum likelihood techniques (Stewart, 1983):

$$y_{i}=X_{i}\beta +\varepsilon_{i}\;iid\;\varepsilon_{i}\;∼\;N(0,\sigma^{2}\;)$$

where *X_i_* is the vector of predictors, *β* is the vector of associated coefficients and *ε_i_* is the error term. From this regression we obtain a point value prediction, $y_{i}^{*}$ that is bounded between the minimum and maximum of hours intervals for individual i:

$$y_{i}^{*}=\max\{m_{i},\;min(y_{i},M_{i})\}$$

To obtain point value provider predictions we use unconfirmed by recipient and confirmed dyads, whereas to obtain point value recipient predictions we use unconfirmed by provider and confirmed dyads. We use the Akaike Information Criteria (AIC) (Akaike, 1974) and the Bay-nesian Information Criteria (BIC) (Stone, 1979) to inform a choice of which set of characteristics to use for the hours prediction. Once we obtain a prediction, we calculate the difference (*π_d_*) between providers’ predicted hours (*p_d_*) and recipients’ predicted hours (*r_d_*) at the dyad level (denoted d) among confirmed dyads:

$$\pi_{d}=p_{d}-r_{d}$$

However, we restrict this calculation to providers who only care for one recipient as additional assumptions would be required to split the total hours between each recipient. We create a binary outcome equal to one if the confirmed dyad’s difference in hours in *π_d_* is in the top or bottom 5th percentiles, and zero otherwise. We use the extended characteristic set and estimate a linear probability model using Ordinary Least Squares.

Obtaining predictions allows provider and recipient dyads time reports to be valued in monetary terms and understand the difference in this valuation between perspectives. Reported weekly hours of care from each perspective and also its difference (*π_d_*) are multiplied by the hourly cost of a replacement home care worker in 2018 at £22 (Curtis and Burns, 2018).

### 3.5 Sample selection

**Fig. 1:**
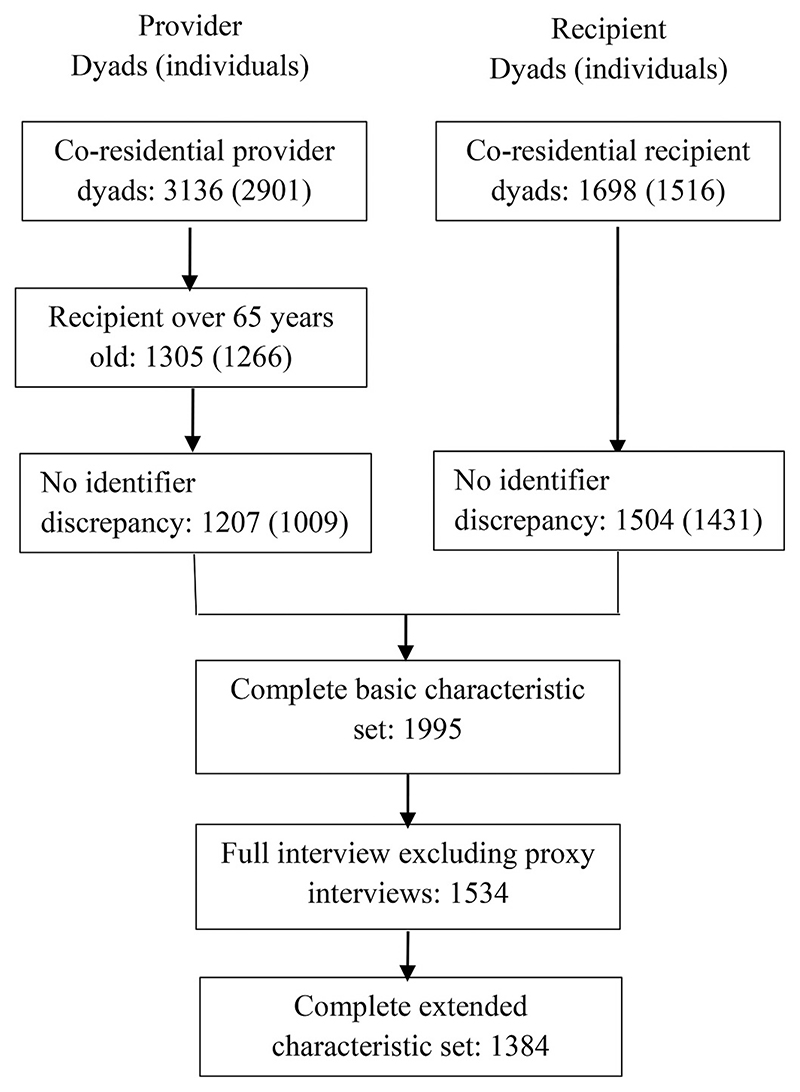
Sample restrictions and derivation.

The restrictions placed on provider and recipient dyads are outlined in Fig. 1, which is among co-residential dyads. From the recipient side we exclude households where there is discrepancy between the relationship and person identifier of the nominated provider. From the provider side, we remove providers who care for someone under 65 years old (as this is the eligibility criteria for receipt questions) and subsequently remove provider dyads in households where the recipient gave conflicting person identifier and relationship information. Provider and recipient datasets combine to obtain 1995 dyads with a complete basic characteristic set. All providers and recipients (both nominated and confirmed) with a full interview, which excludes proxy interviews, obtains 1534 dyads. Our main analysis sample of 1384 dyads includes those with complete cases on the extended characteristics set. For sensitivity analysis, we estimate the multinomial logit using the 1995 and 1534 dyads with the basic set of predictors.

For analysis at the intensive margin, we obtain provider and recipient hours of care predictions from 1259 dyads. This excludes providers who give care to more than one recipient, as there is no information on how provider hours are shared across recipients and exclude those who do not report hours of care provision or receipt. We directly compare the hours between providers and recipients in 404 dyads (out of the 471 confirmed dyads) where both the provider and recipients confirm each other’s reports and thus have associated hour’s reports for comparison.

## 4 Results

### 4.1 Descriptive statistics

Descriptive statistics presented in Table A3 include dyads with complete cases for the extended characteristic set (n = 1384). Recipients are, on average, older than providers across all dyad types. The oldest providers are among confirmed dyads with an average of 71.1 years of age which is reflected in this group having the highest percentage of those retired at 81%. The majority of dyads are spousal as 75% of un-confirmed by recipient dyads, 80% of unconfirmed by provider dyads and 87% of confirmed dyads are of this type. Recipients in confirmed dyads have more restrictions on activities of daily living, on average, compared to unconfirmed dyad types.

### 4.2 Extensive margin results: the level of discrepancy

The most common dyad type in our sample are those which are unconfirmed by the provider at 56.7% (Table 2). There are 9.3% of dyads that are unconfirmed by the recipient and 34% confirmed by both the provider and recipient. Among claims of care provision made by a recipient, 62.5% of these are unconfirmed (the provider under-reported proportion) and 37.5% are confirmed by the nominated provider. Whereas among provider care claims, 21.4% of these are unconfirmed (the recipient under-reported proportion) and 78.7% of these are confirmed by the nominated recipient.

To obtain the possible level of provider and recipient under-reporting across the UK we first estimate that there are 2,472,377 co-residential carers in the UK (based on 38% of 6.5 million carers being co-residential in 2011). We produce an estimate of 5,712,471 caregiving claims, of which 528,403 may be unconfirmed by the recipient and 3,240,114 may be unconfirmed by the provider.

### 4.3 Extensive margin results: predictors

All models are jointly significant as indicated by the statistically significant LR test chi-squared statistic (presented in Table A4). The appropriate estimation strategy given by the Wald test for combining alternatives is to estimate each dyad type separately (p < 0.01 for all combinations). Only the unconfirmed by provider for the extended set and the confirmed dyad type results for ADLs as separate indicators fails the independence of irrelevant alternatives test. The extended predictor set is the preferred specification due to the lower AIC and BIC scores.

Table 3 shows the basic set of predictor results at the extensive margin via the multinomial logit. Recipient age is a strong predictor of dyad type as older recipients are more likely to be in a confirmed dyad and less likely to be in an unconfirmed by provider dyad compared to younger recipients (by —0.7 and 0.8 percentage points respectively for each year of age; p < 0.01). Larger households are less likely to confirm claims of provision and recipient.

Sensitivity results from the samples of 1995 and 1534 dyads show similar results but with more statistical significance (presented in Table A5 and A6, respectively). One notable difference is that the parent-child coefficient is positive and statistically significant in Table A5 but negative (yet statistically insignificant) in Table 3 for un-confirmed recipient dyads.

Table 4 shows the multinomial logit regression estimated with the extended set of predictors. All of the age coefficients tend towards zero with the inclusion of the extended set of characteristics, likely due to health being part of this extended set. Household size remains a strong predictor of agreement between provider and recipients and is the only household level variable to have any statistical significance. Household variables from the extended set (household equivalised income and the number of interviewer calls to the household) and interview characteristics (interview date difference, other present at the interview and whether previously interviewed) have small magnitudes and are not statistically significant at the 5% level.

Providers with a degree qualification are 7.7 (p < 0.01) percentage points more likely to be in agreement with a recipient, relative to providers without a degree qualification. Providers with a health condition or those who receive a carer benefit are less likely (p < 0.05) to leave a recipient’s claim unconfirmed (by 4.5 and 12.9 percentage points, respectively) but more likely (p < 0.05) to have an unconfirmed recipient (by 4.2 and 4.5 percentage points, respectively) compared to providers without a health condition or in receipt of a carer benefit. For each extra activity or instrumental activity of daily living restriction of the recipient, there is more likely to be agreement between the provider and recipient by 5.2 and 9.3 percentage points, respectively.

Results using cross-sectional sample weights separately for the provider (Table A7) and the recipient (Table A8) provided in the UKHLS show a similar magnitude and direction compared to results in Table 4. There is little difference in the coefficients between both sets of weighted results. However, there are some notable exceptions between weighted and unweighted results in terms of the statistical significance and magnitude. These are for the coefficients on female, income and whether the respondent has no previous interview. For example, females are less likely to be in a confirmed dyad but more likely to be in an unconfirmed by provider dyad type. Those with higher household incomes and where both members of the dyad are interviewed on different dates are more likely to be in an unconfirmed by recipient dyad, whereas recipients with no previous interview in the UKHLS are less likely to be in an unconfirmed by recipient dyad.

Results from two separate logits (Table A9) show that recipients reporting larger compared to smaller hours of care receipt have a higher probability of being in a confirmed dyad. For providers this is not the case as coefficients on the hours of provision are small in magnitude and not statistically significant. We conclude that the multinomial logit is the appropriate specification as hours are not predictive of agreement on the recipient side, the multinomial logit offers more information across all dyad types and the evidence against the independence of irrelevant alternatives holding is inconclusive.

Table 5 shows the specification that includes each activity of daily living and instrumental activity of daily living restriction as a separate indicator variable. Among activity of daily living restrictions, if a recipient requires help managing stairs, bathing/showering, getting dressed or taking the right medicine, they are more likely to be a confirmed dyad than recipients who do not require these types of help. In particular, getting dressed and taking the right medicine have the largest magnitudes at 16.5 and 18.1 percentage points respectively. Recipients that require help using the toilet or getting in and out of bed are more likely (by 21.3 and 12.9 percentage points (p < 0.05), respectively) to be in an unconfirmed by recipient dyad than those without these difficulties. Interestingly, the help using the toilet coefficient is negative for confirmed dyads which contrasts with the positive help bathing/showering coefficient.

Among instrumental activity of daily living restrictions, if a recipient requires help with walking down the road, with shopping or with housework they are more likely to be in agreement with their nominated provider by 11.8, 10.7 and 8.6 percentage points (all p < 0.001), respectively than those who do not require these types of help. Recipients who require help with paperwork are 12.3 percentage points (p < 0.001) more likely to be in an unconfirmed by provider dyad and 13.7 percentage points (p < 0.001) less likely to be in an unconfirmed by recipient dyad type than recipients that do not have these difficulties.

### 4.4 Intensive margin results

We chose the extended set of predictors to predict provider and recipient hours due to these models having the lowest AIC and BIC scores (although the BIC statistic was lower for provider models). Full regression output is shown in Table A10. Table 6 shows that among confirmed dyads the providers report 10.56 h per week more, on average, than their recipient does.

**Fig. 2:**
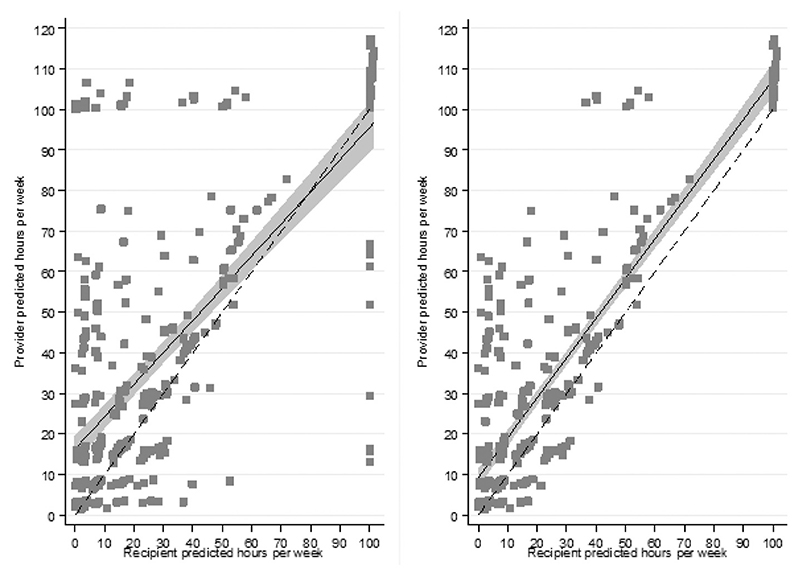
Predicted weekly hours among 404 dyads. *Note: Left side figure includes all dyads and the right side figure removes the top and bottom 5^th^ percentiles. Solid line represents the trend between predicted provider and recipient hours. Shaded region is the 95% confidence interval of the trend

Fig. 2 plots the predicted weekly hours of provider and recipient reports among 404 dyads. The 45-degree line indicates identical hour reports.

We perform a replacement cost calculation that multiplies the average hours of providers and recipients among 404 dyads by the hourly cost of a homecare worker at £22. The average monetary valuation of provider hours per week is £849.64 and for recipients is £617.32. This is a difference of £232.32 per dyad per week in the monetary valuation of hours between provider and recipient reports; a figure that extrapolated to an annual period is £12,080.64.

Table A11 shows results from a linear probability model specification where the outcome is equal to one if the difference between provider and recipient hours is in the top or bottom 5th percentile. For each instrumental activity of daily living restriction of the recipient, a dyad is 3 percentage points more likely to be in the extreme tails of the distribution of differences in reported hours. The models with specific instrumental activity of daily living restrictions included as binary variables show that if the recipient requires help shopping, their dyad will be 12 percentage points more likely to be at the top or bottom 5th percentile of the difference in hours. Recipients with a sight difficulty are 11 percentage points more likely to be in the extremes of the distribution of differences compared to those without a sight difficulty.

## 5 Discussion

### 5.1 Main findings

Informal care research typically only obtains this information from either the provider or recipient perspective. There has been little consideration of whether providers and recipients offer different accounts of caregiving. We find that there is considerable discrepancy between provider and recipient reports of informal caregiving. This is particularly acute at the extensive margin among those who claim to receive informal care as the nominated provider does not confirm 62.5% of these claims but only confirms 37.5% of these claims. Whereas among those who claim to provide informal care the discrepancy is less stark as the nominated recipient does not confirm 21.4% of these claims but confirms 78.7% of these claims. Based on these discrepancies and data from the UK 2011 census, we estimate that there could be 3,240,114 under-reported co-residential care providers if only provider information is used and 528,403 under-reported co-residential recipients if only recipient information is used in the UK.

The scale of carer under-reporting (at 62.5%) relative to recipient under-reporting (at 21.4%) is surprising. Of particular importance is the rising prevalence of dementia, as recipients may not be able to recall that they are in receipt of care, which would threaten the ability to obtain caregiving information from the recipient. We do find that recipients with memory difficulties are more likely to be in an unconfirmed by recipient dyad compared to those without a memory difficulty. On the other hand, how people view caregiving roles may be a factor in explaining the differences in under-reporting proportions. Often those who perform caregiving activities may recognise themselves in terms of their relationship to the recipient, such as a wife or husband, instead of a ‘carer’ (Carduff et al., 2014; Corden and Hirst, 2011; Hughes et al., 2013).

Informal care among particular sub-groups is not accounted for if only one perspective is used. For example, larger households have a higher probability of discrepancy, which may be due to knowledge of responsibilities between individuals within the household. Therefore, to understand caregiving provided within large households both perspectives would be needed to account for the totality of care taking place. Surprisingly, we found no statistically significant evidence that the characteristics of the interview such as others being present and the number of interviewer calls to the household are related to discrepancy. If provider information is used to identify caregiving, carers in relatively worse health (as measured by having a health condition) will be identified compared to those in better health that would be identified via recipients. Thus, health effects of providing care could be over-stated if the provider is used compared to the recipient to collect caregiving information. Activities of daily living and instrumental activities of daily living restrictions are strong predictors of agreement between carers and recipients. Care to those with only a few activities of daily living and instrumental activities of daily living restrictions would not be included if only one perspective is used. If the aim of particular future research were to focus on those who are likely to be intensive carers, then either perspective would likely capture this type of care. However, if the intention is to capture the totality of care, then more consideration is needed to understand what sub-groups may be missed and if these bare relevance on the research objective.

The results from this study confirm that carers may not identify as providing care in cases that involve help with day-to-day tasks they would also do for themselves such as cooking, cleaning and paperwork etc. Whereas caregivers are more likely to identify as such when they provide help with personal care such as help getting dressed and help with stairs. This relates to measurement issues around the distinction between ‘normal’ and ‘caregiving’ tasks (van den Berg et al., 2004).

Our findings at the intensive margin show that providers report more hours than their recipient does. Presented graphically among the confirmed dyads there are clusters of dyads where the provider (recipient) reports the largest possible hours and the recipient (provider) the lowest. This may be plausible to an extent due to question wording. A provider of care can include non-tangible tasks such as social and emotional care as caregiving, due to the open-ended nature of the hours question (Dumont et al., 2010). On the other hand, the hours of care received by a recipient follows on from questions about help with specific daily tasks, not allowing the inclusion of possible non-tangible tasks. Similarly, a recipient may not be aware of the total amount of time a provider dedicates to certain tasks. For example, with help shopping as this was a predictor of large differences between the provider and recipient. Similarly, the UKHLS in wave seven asks about care receipt hours in the past week but asks about care provision hours in a typical week.

The monetary value difference of £278 per week, on average, within confirmed dyads is substantial as this makes up roughly a quarter of the monetary value of provider hours. This may mean that even if both provider and recipient perspectives are used to identify caregiving at the extensive margins, the recipient hours from unconfirmed dyads may be smaller than the unattained provider hours. Consideration of this must be made when including informal care in an economic evaluation as this may impact on the cost effectiveness of an intervention (Goodrich et al., 2012; Krol et al., 2015). As a result, using hours from another perspective as a sensitivity check would be advisable given that visual inspection showed extremes in the differences between hourly reports in some cases.

Our result of provider under-reporting at 62% is larger than the 47% found by Rutherford and Bu (2018). However, the recipient under-reporting proportion we find at 22% is closer, although larger, than the 19% reported by Rutherford and Bu (2018). They also find, similar to the present study, that the recipient’s health condition and activity of daily living restrictions are associated with provider under-reporting. Although, in contrast they did not find that memory difficulties were related to under-reporting. The difference in question wording between ELSA and UKHLS may account for the contrast in discrepancies at both margins although in both surveys the recipient questions are based on ADL and IADL tasks. The provider side, although worded differently to the UKHLS, does have a similarity to the UKHLS as it refers to caring as ‘looking after’ at the extensive margin. At the intensive margin, Rutherford and Bu (2018) report no information on the magnitude of difference between provider and recipient hour reports, they found 46% of carers reported larger hours and 10% fewer hours than recipients. This complements the larger hours we found for carers compared to recipients. A possible explanation for differences in our results to Rutherford and Bu (2018) at both margins may be that our study included more than spousal dyad types.

### 5.2 Strengths and limitations

A major strength of this study is the consideration of the extensive and intensive margins of discrepancy. The UKHLS has a rich set of information, enabling a range of predictors to be analysed at both margins. We also show that even among confirmed dyads, there are substantial differences in reported hours of care between providers and recipients. Our analysis furthers the literature on this area by distinguishing between two types of unconfirmed dyads using a multinomial logit specification. This provides more detail on particular sub-groups of carers and recipients that could be missed if only one perspective is used.

These discrepancies may only arise due to the structure and wording of the informal care questions in the UKHLS. On the provider side, the caregiving question is restricted in the sense that it gives the respondent only one opportunity to indicate they are a carer, but the question is open to the respondent’s interpretation of what they constitute as ‘looking after’ or ‘giving special help to’ and who they judge to need this as a result of being ‘sick, disabled and elderly’. On the recipient side, the questions are broader given that a respondent can indicate help with a variety of ADL and IADL tasks. Although these questions are also restrictive as they have a more convoluted question routing as part of the social care module and they do not allow a respondent to indicate they received supervision or emotional support. Based on our findings, it would appear that indicating the tasks of caregiving could lead to a greater identification of caregiving taking place.

A further limitation is the generalisability of results. Our results relate to carers and recipients who have completed the full questionnaire, conditional on the recipient being over 65 years old and co-residing with the provider. We expect that those who have filled in the questionnaire have better health than those who have not. However, we show that results from a larger sample with unconfirmed by provider and by recipient dyads who did not complete the full questionnaire are broadly similar to our main results. Those 65 years old and over represent the age group which requires the most caregiving. Co-residential caregiving is likely to have higher discrepancy proportions than extra-residential care as spousal dyads may find it more difficult to separate ‘normal’ and ‘caregiving’ activities (van den Berg et al., 2004). We do not consider the totality of caregiving hours given by a provider who has more than one recipient, due to the difficulty of splitting hours between recipients. Although providers with more than one recipient were far less common than those who reported one recipient. Future research may be able to consider both intensive and extensive margins combined if in a particular survey follow-up is possible to unconfirmed providers or recipients.

Estimates on the scale of under-reporting of caregiving in the UK rely upon the UKHLS being representative of caregiving in the UK and the level of informal care to have remained constant since 2011. These figures would only be valid for the provision and receipt questions found in the survey. Other types of questions could result in different out-comes. Further, the census is person level, not dyad level. Therefore our figures should be treated with caution but nonetheless provide some indication on the scale of under-reporting of caregiving in the UK. Similarly, our analysis focuses on under-reporting as opposed to over-reporting, as the latter may occur in some instances. Although given a focus on possible reasons for under-reporting in the literature (Carduff et al., 2014; Corden and Hirst, 2011; Grande et al., 1997) this suggests under-reporting may be more likely than over-reporting.

## 6 Conclusions

There is no gold standard claim regarding how to collect informal care information in terms of: which perspective to use (such as the provider or recipient), question wording, question routing, the optimal recall period and the type of tasks to include. There is a consensus to reflect the heterogeneous nature of informal caregiving when including these types of questions (Rutherford and Bu, 2018; van den Berg et al 2004; van den Berg and Spauwen, 2006). However, a lack of specificity on what is classified as caregiving can prohibit comparability between studies, for example, with the inclusion or exclusion of intangible aspects (such as providing social and emotional care) of caregiving (Dumont et al., 2010). Nevertheless, whether to obtain informal care information from the provider or recipient is likely to be one of the first measurement considerations of a researcher.

Numerous studies have used informal care information, but there has been little consideration of analysis from alternative perspectives. Our findings demonstrate the importance of understanding the supply of a non-market good through accounts of the parties involved in the exchange. Most importantly, as each perspective is likely to result in different sub-groups indicating informal care is taking place. Ideally, it would be preferable to combine provider and recipient accounts for informal care related research questions, if possible. Alternatively, the results provided in this study can assist researchers in understanding which sub-groups may be under-represented if they use one perspective of caregiving and the likely impact of this on the research objective. Future research could explore this discrepancy in a panel data context to explore whether discrepancy proportions are consistent within the same dyads over time.

## Supplementary Material

A

## Figures and Tables

**Table 1:** Informal care dyad types in terms of correspondence between reports.

Dyad Type	Informal care	
	Provider	Recipient
Confirmed - PR (1-1)	✓	✓
Unconfirmed by recipient - R (1-0)	✓	X
Unconfirmed by provider - P (0-1)	X	✓
No claims - N (0-0)	X	X

**Table 2:** Discrepancy in informal care provider and recipient reports.

	Dyad type:		
	Unconfirmed by Recipient	Unconfirmed by Provider	Confirmed
n %	128 (9.25)	785 (56.72)	471 (34.03)
Under reported proportion^a^	*21.37*	*62.50*	
England and Wales level n	528,403	3,240,114	1,943,954

a *Note:* Based on 1384 dyads where both individuals in the dyad have completed a full interview and have complete cases for the extended set of characteristics. The under-reported percentage for recipients is calculated as $\frac{\sum_{d}R}{\sum_{d}R+\sum_{d}PR}$ using the number of unconfirmed recipient and confirmed dyads. The under-reported proportion for providers is calculated as $\frac{\sum_{d}P}{\sum_{d}P+\sum_{d}PR}$ using the number of unconfirmed provider confirmed dyads.

**Table 3:** Average marginal effects from the multinomial specification estimated using the basic set of characteristics.

	Dyad Type					
	Unconfirmed Recipient		Unconfirmed Provider		Confirmed	
	Marginal eff	Std err	Marginal eff	Std err	Marginal eff	Std err
**Provider variables:**						
Female	0.018	(0.021)	0.014	(0.063)	–0.032	(0.064)
Age	–0.001	(0.001)	0.004*	(0.002)	–0.003	(0.002)
**Recipient variables:**						
Female	0.006	(0.023)	0.064	(0.065)	–0.070	(0.066)
Age	–0.001	(0.001)	–0.007**	(0.002)	0.008***	(0.002)
**Dyad variables:**						
Parent-Child	–0.005	(0.037)	–0.079	(0.073)	0.083	(0.075)
Other	0.102**	(0.036)	–0.048	(0.091)	–0.054	(0.082)
**Household variables:**						
Household size	0.061**	(0.020)	0.652***	(0.069)	–0.712***	(0.081)
Wales	0.031	(0.033)	–0.072	(0.047)	0.041	(0.044)
Scotland	0.004	(0.032)	–0.035	(0.051)	0.031	(0.049)
Northern Ireland	–0.029	(0.025)	0.053	(0.049)	–0.023	(0.045)
N		1384				
Household		1181				

*Note:* Base categories for female, dyads and region variables, are male, spousal dyad and England, respectively. Standard errors clustered at the household level in parentheses: *p < 0.05; **p < 0.01, ***p < 0.001.

**Table 4:** Average marginal effects from the multinomial specification estimated using the extended set of characteristics.

	Dyad Type					
	Unconfirmed Recipient		Unconfirmed Provider		Confirmed	
	Marginal eff	Std err	Marginal eff	Std err	Marginal eff	Std err
**Basic predictor set:**						
**Provider variables:**						
Female	–0.000	(0.021)	0.023	(0.062)	–0.023	(0.059)
Age	–0.001	(0.001)	0.003	(0.002)	–0.002	(0.002)
**Recipient variables:**						
Female	0.004	(0.021)	0.060	(0.063)	–0.064	(0.060)
Age	0.002	(0.002)	–0.002	(0.002)	0.000	(0.002)
**Dyad variables:**						
Parent-Child	–0.011	(0.037)	–0.085	(0.068)	0.096	(0.069)
Other	0.075*	(0.035)	–0.056	(0.103)	–0.018	(0.092)
**Household variables:**						
Household size	0.066***	(0.016)	0.706***	(0.076)	–0.772***	(0.085)
Wales	0.052	(0.032)	–0.080	(0.044)	0.028	(0.039)
Scotland	0.006	(0.029)	–0.008	(0.046)	0.001	(0.041)
Northern Ireland	0.005	(0.029)	0.057	(0.046)	–0.062	(0.039)
**Extended predictor set:**						
**Provider variables:**						
Ethnic group: UK	–0.021	(0.028)	0.028	(0.051)	–0.007	(0.050)
Degree qualification	–0.022	(0.018)	–0.055*	(0.027)	0.077**	(0.025)
Retired	–0.034	(0.025)	0.039	(0.039)	–0.005	(0.035)
Health condition	0.042**	(0.014)	–0.045*	(0.022)	0.003	(0.019)
Carer benefit	0.075*	(0.030)	–0.129*	(0.057)	0.054	(0.051)
Others present	–0.004	(0.016)	–0.005	(0.024)	0.009	(0.022)
No previous interview	0.006	(0.080)	0.042	(0.084)	–0.048	(0.092)
**Recipient variables:**						
Ethnic group: UK	0.020	(0.033)	–0.141*	(0.056)	0.122*	(0.055)
Degree qualification	0.024	(0.018)	0.004	(0.029)	–0.029	(0.027)
Number of ADLs	–0.039	(0.022)	–0.013	(0.017)	0.052***	(0.012)
Number of IADLs	–0.076***	(0.012)	–0.017	(0.013)	0.093***	(0.008)
Memory difficulty	0.054**	(0.019)	–0.169***	(0.033)	0.116***	(0.028)
Sight difficulty	0.059*	(0.028)	–0.139***	(0.040)	0.080*	(0.036)
Recipient benefit	0.046*	(0.022)	–0.116**	(0.036)	0.070*	(0.033)
Others present	–0.025	(0.016)	-0.013	(0.025)	0.038	(0.023)
No previous interview	–0.138	(0.081)	0.021	(0.091)	0.117	(0.080)
**Dyad variables:**						
Interview date difference	0.016	(0.021)	0.007	(0.043)	–0.023	(0.040)
**Household variables:**						
Income (000’s)	0.009	(0.006)	–0.000	(0.012)	–0.008	(0.010)
Calls to household	–0.000	(0.002)	0.000	(0.004)	–0.000	(0.004)
N		1384				
Household		1181				

*Note:* Base categories for female, dyads and region variables, are male, spousal dyad and England, respectively. Standard errors clustered at the household level in parentheses: *p < 0.05; **p < 0.01, ***p < 0.001.

**Table 5:** Average marginal effects of instrumental activities of daily living and activities of daily living from the multinomial specification estimated using the extended set of characteristics.

	Dyad Type					
	Unconfirmed Recipient		Unconfirmed Provider		Confirmed	
	Marginal eff	Std err	Marginal eff	Std err	Marginal eff	Std err
**Activities of daily living variables:**						
Help managing stairs	–0.099*	(0.041)	0.005	(0.045)	0.093**	(0.032)
Help getting around the house	0.077	(0.070)	–0.010	(0.078)	–0.067	(0.059)
Help getting in/out bed	0.129*	(0.050)	–0.087	(0.066)	–0.042	(0.069)
Help cutting toenails	–0.093***	(0.013)	0.048*	(0.022)	0.045*	(0.020)
Help bathing/showering	–0.024	(0.043)	–0.071	(0.049)	0.096*	(0.041)
Help using the toilet	0.213*	(0.088)	–0.025	(0.105)	–0.188*	(0.091)
Help eating	–0.040	(0.141)	0.030	(0.130)	0.010	(0.068)
Help washing	0.042	(0.132)	0.129	(0.151)	–0.171	(0.134)
Help getting dressed	–0.090*	(0.040)	–0.075	(0.044)	0.165***	(0.034)
Help taking medicine	–0.089*	(0.039)	–0.092	(0.048)	0.181***	(0.033)
**Instrumental activities of daily living variables:**						
Help walking down the road	0.023	(0.027)	–0.141***	(0.032)	0.118***	(0.025)
Help shopping	–0.108***	(0.015)	0.001	(0.025)	0.107***	(0.022)
Help with housework	–0.085***	(0.019)	–0.000	(0.026)	0.086***	(0.022)
Help with paperwork	–0.137***	(0.019)	0.123***	(0.023)	0.014	(0.020)
N		1384				
Households		1181				

*Note:* Includes the extended set of predictors. Standard errors are clustered at the household level in parentheses: *p < 0.05; **p < 0.01, ***p < 0.001.

**Table 6:** Predicted hours of providers and recipients.

	Basic set		Extended set	
	Provider	Recipient	Provider	Recipient
**Unconfirmed and confirmed dyad types:**				
Mean predicted weekly hours (SD)	36.2 (35.5)	14.6 (25.0)	36.8 (36.5)	14.9 (25.4)
N	528	1200	528	1200
AIC	2722.3	8133.5	2659.5	7656.9
BIC	2773.6	8194.6	2791.8	7814.7
**Confirmed dyads:**				
Mean predicted weekly hours (SD)	37.8 (35.4)	27.2 (32.8)	38.6 (36.5)	28.1 (33.0)
Difference (SD)	10.6(27.1)		10.6(27.1)	
Monetary value in £	831.60	597.74	849.64	617.32
Difference in monetary value in £	233.86		232.32	
N	404	404	404	404
