# Supplementary material for: The extent and predictors of discrepancy between provider and recipient reports of informal caregiving
Source: Soc Sci Med. Author manuscript; Available in PMC 2022 Jul 20. (PMC7613109; doi:10.1016/j.socscimed.2021.113890)
Supplement: A [file EMS149411-supplement-A.docx]

## **Appendix**

Table A1: Predictor sets

| Level | Variable | Description |
| --- | --- | --- |
|  |  |  |
| **Basic characteristics set from first contact:** | | |
| Provider | Age | Age in years of provider |
| Recipient | Age | Age in years of recipient |
| Provider | Female | =1 if provider is female |
| Recipient | Female | =1 if recipient is female |
| Dyad | Dyad type | =1 if dyad is spousal |
| Dyad | Dyad type | =1 if dyad is an adult child and parent |
| Dyad | Dyad type | =1 if dyad is other relationship |
| Household | Household size | Number of individuals living in the household |
| Household | Region | =1 if household is in Wales |
| Household | Region | =1 if household is in Scotland |
| Household | Region | =1 if household is in Northern Ireland |
|  | | |
| **Extended characteristics set from the full interview:** | | |
| Provider | Ethnic group: UK | =1 if ethnic group is British/English/Scottish/Welsh/Northern Irish |
| Recipient | Ethnic group: UK | =1 if ethnic group is British/English/Scottish/Welsh/Northern Irish |
| Provider | Degree qualification | =1 if highest qualification is at degree level |
| Recipient | Degree qualification | =1 if highest qualification is at degree level |
| Provider | Retired | =1 if retired from the labour force |
| Recipient | Number of ADLs | Number of ADLs ranging from 0-3 |
| Recipient | Number of IADLs | Number of IADLs ranging 0-11 |
| Provider | Has a health condition | =1 if the provider has a health condition |
| Recipient | Memory difficulty | =1 if the recipient has a memory difficulty |
| Recipient | Sight difficulty | =1 if the recipient has a sight difficulty |
| Provider | Carer benefit | =1 if the provider claims carer allowance |
| Recipient | Recipient benefit | =1 if the recipient claims attendance allowance |
| Provider | Others present | =1 if anyone is present during the provider interview |
| Recipient | Others present | =1 if anyone is present during the recipient interview |
| Provider | Not interviewed before | =1 if not interviewed in previous survey waves |
| Recipient | Not interviewed before | =1 if not interviewed in previous survey waves |
| Dyad | Dyad interview date difference | =1 if both dyad members interviewed on different days |
| Household | Household Income (000’s) | Equivalised monthly household income using OECD scale |
| Household | Interviewer calls to households | Number of times an interviewer has contacted household |
| *Note:* Ethnic group question has the same categories as used in the 2011 England and Wales census. ADL – Activities of daily living. IADL – Instrumental activities of daily living. OECD - Organisation for Economic Co-operation and Development | | |

Table A2: Assigned upper and lower bounds to informal care provision and receipt hour reports

| Category | Lower-bound ($m_{i})$ | Upper-bound ${(M}_{i})$ |
| --- | --- | --- |
| **Provision:** |  |  |
| 0-4 | 1 | 4 |
| 5-9 | 5 | 9 |
| 10-19 | 10 | 19 |
| 20-34 | 20 | 34 |
| 35-49 | 35 | 49 |
| 50-99 | 50 | 99 |
| 100+ | 100 | 168 |
| Varies under 20 | 0 | 20 |
| Varies over 20 | 20 | 30 |
| Some other times | 2 | 2 |
| **Receipt:** |  |  |
| No help in the past week | 0 | 0 |
| Less than one hour | 0 | 1 |
| 1-4 | 1 | 4 |
| 5-9 | 5 | 9 |
| 10-19 | 10 | 19 |
| 20-34 | 20 | 34 |
| 35-49 | 35 | 49 |
| 50-99 | 50 | 99 |
| 100+ | 100 | 168 |

Table A3: Descriptive statistics of respondent characteristics by dyad type

| Dyad Type: | | | | | | | | |
| --- | --- | --- | --- | --- | --- | --- | --- | --- |
|  | Unconfirmed by recipient (n=128) | | Unconfirmed by provider | | | Confirmed | | |
|  | Unconfirmed | | Unconfirmed | | | Confirmed | Confirmed | |
|  | Provider  Provider | Recipient (SD) | Provider (SD) | Recipient (SD) | | Provider (SD) | Recipient (SD) | |
| Mean (SD) | | | | | | | | |
| **Basic set:** | | | | | | | | |
| Female | 0.55 | 0.51 | 0.50 | | 0.55 | 0.53 | | 0.49 |
| Age | 65.7 (14.7) | 74.7(7.4) | 67.4(14.4) | | 74.9(6.8) | 71.1 (11.1) | | 76.4(7.1) |
| Spousal dyad | 0.75 | | 0.80 | | | 0.87 | | |
| Parent child dyad | 0.13 | | 0.15 | | | 0.10 | | |
| Other dyad | 0.12 | | 0.05 | | | 0.03 | | |
| Household size (SD) | 2.3(0.8) | | 2.6(1.1) | | | 2.0(0.1) | | |
| England | 0.77 | | 0.79 | | | 0.76 | | |
| Wales | 0.11 | | 0.08 | | | 0.10 | | |
| Scotland | 0.07 | | 0.06 | | | 0.07 | | |
| Northern Ireland | 0.05 | | 0.07 | | | 0.07 | | |
| **Extended set:** | | | | | | | | |
| Ethnic Group: UK group:UK | 0.88 | 0.90 | 0.85 | | 0.85 | 0.94 | | 0.95 |
| Degree qualification | 0.24 | 0.23 | 0.23 | | 0.16 | 0.23 | | 0.14 |
| Retired | 0.64 | - | 0.73 | | - | 0.81 | | - |
| # ADLs (SD) | - | 0.6(1.4) | - | | 1.3(1.8) | - | | 2.3(2.2) |
| # IADLs (SD) | - | 0.6(1.2) | - | | 1.7(1.2) | - | | 2.5(1.3) |
| Health condition | 0.65 | - | 0.51 | | - | 0.56 | | - |
| Memory difficulty | - | 0.18 | - | | 0.11 | - | | 0.22 |
| Sight difficulty | - | 0.10 | - | | 0.07 | - | | 0.16 |
| Carer benefit | 0.10 | - | 0.04 | | - | 0.09 | | - |
| Recipient benefit | - | 0.13 | - | | 0.09 | - | | 0.21 |
| Others at interview | 0.41 | 0.38 | 0.48 | | 0.50 | 0.60 | | 0.63 |
| Not interviewed before | 0.02 | 0.01 | 0.02 | | 0.02 | 0.01 | | 0.02 |
| Interview date difference | 0.13 | | 0.10 | | | 0.05 | | |
| Household income (SD) | 2.0(1.4) | | 1.7(1.0) | | | 1.6(0.9) | | |
| # Calls to household (SD) | 4.1(2.5) | | 4.2(3.0) | | | 3.7(2.3) | | |

| Table A4: Specification tests | | | |
| --- | --- | --- | --- |
|  | Basic predictor set | Extended predictor set | |
|  |  | ADLs as counts | ADLs as separate binary indicators |
| McFadden R-squared | 0.124 | 0.332 | 0.400 |
| AIC | 2248.110 | 1800.673 | 1005.21 |
| BIC | 2363.230 (df=22) | 2114.637 (df=54) | 2117.424 (df=82) |
| LR test of model significance (χ^2^) | 310.98*** (df=20) | 834.41*** (df=58) | 390.790*** (df=82) |
| Wald test for combining alternatives (χ^2^): | |  |  |
| Type R & P | 27.071** (df=10) | 122.417*** (df=31) | 146.771*** (df=41) |
| Type P & PR | 58.775*** (df=10) | 231.519*** (df=29) | 259.656*** (df=41) |
| Type R & PR | 99.923*** (df=10) | 233.877*** (df=29) | 264.580*** (df=41) |
| Hausman-Mcfadden IIA test (χ^2^): | |  |  |
| Unconfirmed by recipient | 11.114 (df=11) | 35.803 (df=32) | 31.104 (df=42) |
| Unconfirmed by provider | 14.894 (df=11) | 92.296*** (df=32) | 104.853*** (df=42) |
| Confirmed | 7.599 (df=11) | 29.039 (df=32) | 29.142 (df=42) |
| Small-Hsiao IIA test (χ^2^) : |  |  |  |
| Unconfirmed by recipient | 10.163 (df=11) | 29.622 (df=30) | 52.031 (df=42) |
| Unconfirmed by provider | 8.034 (df=11) | 30.397 (df=30) | 40.701 (df=42) |
| Confirmed | 14.482 (df=11) | 39.714 (df=30) | 70.881** (df=42) |
| N | 1384 | 1384 | 1384 |
| Households | 1181 | 1181 | 1181 |
| *Note:* *p<0.05; **p<0.01, ***p<0.001. df – degrees of freedom | | | |
|  |  |  |  |

| Table A5: Average marginal effects from the multinomial specification estimated using the basic set of characteristics among 1995 dyads | | | | | | |
| --- | --- | --- | --- | --- | --- | --- |
|  | Unconfirmed Recipient | | Unconfirmed Provider | | Confirmed | |
|  | Marginal eff | Std err | Marginal eff | Std err | Marginal eff | Std err |
| **Provider variables:** | | | | | | |
| Female | 0.041 | (0.022) | -0.027 | (0.038) | -0.015 | (0.041) |
| Age | -0.000 | (0.001) | 0.002 | (0.002) | -0.001 | (0.001) |
| **Recipient variables:** | | | | | | |
| Female | 0.009 | (0.025) | 0.020 | (0.041) | -0.030 | (0.042) |
| Age | 0.005** | (0.002) | -0.010*** | (0.002) | 0.004* | (0.002) |
| **Dyad variables:** | | | | | | |
| Parent | 0.106** | (0.039) | -0.114* | (0.058) | 0.008 | (0.056) |
| Other | 0.231*** | (0.042) | -0.165* | (0.077) | -0.066 | (0.067) |
| **Household variables:** | | | | | | |
| Household size | 0.145*** | (0.017) | 0.368*** | (0.043) | -0.513*** | (0.056) |
| Wales | 0.090* | (0.037) | -0.102* | (0.040) | 0.012 | (0.033) |
| Scotland | -0.021 | (0.040) | -0.005 | (0.048) | 0.026 | (0.040) |
| Northern Ireland | -0.016 | (0.041) | 0.012 | (0.048) | 0.004 | (0.034) |
| N  Households  Wald chi-sq (df=20)  Adjusted McFadden R-Squared  AIC  BIC (dF=22) | | 1995 |  |  |  |  |
|  |  | 1647 |  |  |  |  |
|  |  | 240.881*** |  |  |  |  |
|  |  | 0.128 |  |  |  |  |
|  |  | 3649.010 |  |  |  |  |
|  |  | 3772.175 |  |  |  |  |
| Wald test for combining alternatives: | | | |  |  |  |
| Type R & P chi-sq (df=10) | | 141.645*** | |  |  |  |
| Type P & PR chi-sq (df=10) | | 101.621*** | |  |  |  |
| Type R & PR chi-sq(df=10) | | 104.850*** | |  |  |  |
| *Note:* Base categories for female, dyads and region variables, are male, spousal dyad and England, respectively. Standard errors clustered at the household level in parentheses: *p<0.05; **p<0.01, ***p<0.001 | | | | | | |

| Table A6: Average marginal effects from the multinomial specification estimated using the basic set of characteristics among 1534 dyads | | | | | | |
| --- | --- | --- | --- | --- | --- | --- |
|  | Unconfirmed Recipient | | Unconfirmed Provider | | Confirmed | |
|  | Marginal eff | Std err | Marginal eff | Std err | Marginal eff | Std err |
| **Provider variables:** | | | | | | |
| Female | -0.001 | (0.022) | 0.008 | (0.057) | -0.006 | (0.059) |
| Age | -0.001 | (0.001) | 0.005** | (0.002) | -0.003 | (0.002) |
| **Recipient variables:** | | | | | | |
| Female | -0.002 | (0.023) | 0.039 | (0.060) | -0.037 | (0.062) |
| Age | -0.000 | (0.001) | -0.009*** | (0.002) | 0.009*** | (0.002) |
| **Dyad variables:** | | | | | | |
| Parent | -0.011 | (0.035) | -0.038 | (0.072) | 0.049 | (0.074) |
| Other | 0.098** | (0.036) | -0.020 | (0.083) | -0.079 | (0.077) |
| **Household variables:** | | | | | | |
| Household size | 0.065*** | (0.016) | 0.541*** | (0.057) | -0.605*** | (0.066) |
| Wales | 0.029 | (0.033) | -0.075 | (0.045) | 0.047 | (0.042) |
| Scotland | 0.003 | (0.032) | -0.045 | (0.051) | 0.043 | (0.048) |
| Northern Ireland | -0.022 | (0.026) | 0.035 | (0.046) | -0.013 | (0.041) |
| N  Households  Wald chi-sq (df=20)  Adjusted McFadden RSquared  AIC | | 1534 |  |  |  |  |
|  |  | 1298 |  |  |  |  |
|  |  | 144.656*** |  |  |  |  |
|  |  | 0.103 |  |  |  |  |
|  |  | 2532.460 |  |  |  |  |
| BIC (df=54) | | 2649.843 |  |  |  |  |
| Wald test for combining alternatives: | | | |  |  |  |
| Type R & P chi-sq (df=10) | | 33.224*** | |  |  |  |
| Type P & PR chi-sq (df=10) | | 57.798*** | |  |  |  |
| Type R & PR chi-sq(df=10) | | 109.637*** | |  |  |  |
| *Note:* Base categories for female, dyads and region variables, are male, spousal dyad and England, respectively. Standard errors clustered at the household level in parentheses: *p<0.05; **p<0.01, ***p<0.001 | | | | | | |

Table A7: Average marginal effects from the multinomial specification estimated using the extended set of characteristics (provider weighted)

|  | Dyad type: | | | | | |
| --- | --- | --- | --- | --- | --- | --- |
|  | Unconfirmed by Recipient | | Unconfirmed by Provider | | Confirmed | |
|  | Marginal eff | Std err | Marginal eff | Std err | Marginal eff | Std err |
| **Basic predictor set:** | | | | | | |
| **Provider variables:** | | | | | | |
| Female | -0.021 | (0.027) | 0.166* | (0.080) | -0.145 | (0.076) |
| Age | -0.000 | (0.001) | 0.003 | (0.002) | -0.003 | (0.002) |
| **Recipient variables:** | | | | | | |
| Female | -0.020 | (0.028) | 0.212** | (0.082) | -0.192* | (0.078) |
| Age | 0.002 | (0.002) | -0.002 | (0.003) | -0.000 | (0.003) |
| **Dyad variables:** | | | | | | |
| Parent-Child | -0.015 | (0.039) | -0.119 | (0.090) | 0.134 | (0.086) |
| Other | 0.067 | (0.056) | -0.137 | (0.108) | 0.070 | (0.092) |
| **Household variables:** | | | | | | |
| Household size | 0.070*** | (0.021) | 0.715*** | (0.089) | -0.785*** | (0.101) |
| Wales | 0.068 | (0.039) | -0.087 | (0.048) | 0.019 | (0.043) |
| Scotland | 0.006 | (0.033) | -0.013 | (0.052) | 0.008 | (0.046) |
| Northern Ireland | 0.015 | (0.037) | 0.073 | (0.054) | -0.089 | (0.047) |
| **Extended predictor set:** | | | | | | |
| **Provider variables:** | |  |  |  |  |  |
| Ethnic group: UK | 0.009 | (0.035) | 0.068 | (0.073) | -0.077 | (0.067) |
| Degree qualification | -0.038 | (0.022) | -0.035 | (0.032) | 0.072* | (0.030) |
| Retired | -0.037 | (0.032) | 0.056 | (0.047) | -0.019 | (0.042) |
| Health condition | 0.047** | (0.017) | -0.054* | (0.025) | 0.007 | (0.023) |
| Carer benefit | 0.066 | (0.035) | -0.105 | (0.064) | 0.038 | (0.057) |
| Others present | -0.018 | (0.020) | 0.004 | (0.029) | 0.014 | (0.027) |
| No previous interview | -0.018 | (0.062) | 0.309* | (0.155) | -0.291 | (0.168) |
| **Recipient variables:** | |  |  |  |  |  |
| Ethnic group: UK | -0.006 | (0.041) | -0.160 | (0.090) | 0.167 | (0.086) |
| Degree qualification | 0.033 | (0.021) | 0.010 | (0.034) | -0.042 | (0.032) |
| Number of ADLs | -0.022 | (0.024) | -0.031 | (0.017) | 0.052*** | (0.014) |
| Number of IADLs | -0.079*** | (0.013) | -0.018 | (0.014) | 0.097*** | (0.010) |
| Memory difficulty | 0.049* | (0.020) | -0.182*** | (0.036) | 0.133*** | (0.032) |
| Sight difficulty | 0.045 | (0.030) | -0.175*** | (0.046) | 0.130** | (0.041) |
| Recipient benefit | 0.037 | (0.024) | -0.083 | (0.043) | 0.046 | (0.040) |
| Others present | -0.010 | (0.020) | -0.019 | (0.029) | 0.029 | (0.028) |
| No previous interview | -0.042 | (0.073) | 0.125 | (0.125) | -0.083 | (0.118) |
| **Dyad variables:** |  |  |  |  |  |  |
| Interview date difference | 0.040 | (0.024) | -0.015 | (0.055) | -0.025 | (0.049) |
| **Household variables:** | |  |  |  |  |  |
| Income (000’s) | 0.019** | (0.006) | 0.001 | (0.014) | -0.021 | (0.012) |
| Calls to household | -0.002 | (0.003) | 0.000 | (0.005) | 0.002 | (0.005) |
| N  Households  Wald chi-sq (df=20)  Adjusted McFadden R-Squared  Log likelihood: Model  Log likelihood: Intercept Only | | 1301 |  |  |  |  |
| Household | | 1169 |  |  |  |  |
| *Note:* Base categories for female, dyads and region variables, are male, spousal dyad and England, respectively. The sample is smaller relative to 1384 in the main analysis sample as some respondents have a zero weight by design. Standard errors clustered at the household level in parentheses: *p<0.05; **p<0.01, ***p<0.001 | | | | | | |

Table A8: Average marginal effects from the multinomial specification estimated using the extended set of characteristics (recipient weighted)

|  | Dyad type: | | | | | |
| --- | --- | --- | --- | --- | --- | --- |
|  | Unconfirmed by Recipient | | Unconfirmed by Provider | | Confirmed | |
|  | Marginal eff | Std err | Marginal eff | Std err | Marginal eff | Std err |
| **Basic predictor set:** | | | | | | |
| **Provider variables:** | | | | | | |
| Female | -0.014 | (0.027) | 0.170* | (0.082) | -0.156* | (0.079) |
| Age | 0.000 | (0.001) | 0.003 | (0.002) | -0.003 | (0.002) |
| **Recipient variables:** | | | | | | |
| Female | -0.017 | (0.028) | 0.219** | (0.084) | -0.202* | (0.082) |
| Age | 0.002 | (0.002) | -0.002 | (0.003) | 0.000 | (0.003) |
| **Dyad variables:** | | | | | | |
| Parent-Child | -0.013 | (0.039) | -0.135 | (0.090) | 0.147 | (0.086) |
| Other | 0.064 | (0.053) | -0.124 | (0.110) | 0.060 | (0.097) |
| **Household variables:** | | | | | | |
| Household size | 0.074** | (0.022) | 0.741*** | (0.092) | -0.814*** | (0.105) |
| Wales | 0.057 | (0.038) | -0.083 | (0.048) | 0.026 | (0.044) |
| Scotland | 0.001 | (0.032) | -0.009 | (0.052) | 0.008 | (0.046) |
| Northern Ireland | 0.013 | (0.035) | 0.071 | (0.053) | -0.084 | (0.047) |
| **Extended predictor set:** | | | | | | |
| **Provider variables:** | |  |  |  |  |  |
| Ethnic group: UK | 0.004 | (0.034) | 0.033 | (0.071) | -0.038 | (0.064) |
| Degree qualification | -0.029 | (0.022) | -0.039 | (0.032) | 0.068* | (0.030) |
| Retired | -0.034 | (0.032) | 0.048 | (0.047) | -0.014 | (0.042) |
| Health condition | 0.052** | (0.017) | -0.053* | (0.025) | 0.001 | (0.023) |
| Carer benefit | 0.074* | (0.034) | -0.106 | (0.063) | 0.031 | (0.056) |
| Others present | -0.021 | (0.020) | 0.008 | (0.029) | 0.013 | (0.027) |
| No previous interview | -0.046 | (0.084) | 0.332* | (0.155) | -0.286 | (0.168) |
| **Recipient variables:** | |  |  |  |  |  |
| Ethnic group: UK | 0.025 | (0.041) | -0.124 | (0.081) | 0.099 | (0.077) |
| Degree qualification | 0.021 | (0.021) | 0.019 | (0.034) | -0.041 | (0.032) |
| Number of ADLs | -0.019 | (0.024) | -0.034 | (0.017) | 0.052*** | (0.014) |
| Number of IADLs | -0.077*** | (0.014) | -0.021 | (0.014) | 0.098*** | (0.010) |
| Memory difficulty | 0.043* | (0.021) | -0.181*** | (0.035) | 0.137*** | (0.032) |
| Sight difficulty | 0.058 | (0.031) | -0.190*** | (0.046) | 0.132** | (0.041) |
| Recipient benefit | 0.038 | (0.024) | -0.097* | (0.042) | 0.059 | (0.039) |
| Others present | -0.010 | (0.019) | -0.018 | (0.029) | 0.029 | (0.027) |
| No previous interview | -0.202* | (0.089) | 0.245 | (0.133) | -0.043 | (0.122) |
| **Dyad variables:** |  |  |  |  |  |  |
| Interview date difference | 0.049* | (0.025) | -0.029 | (0.056) | -0.020 | (0.050) |
| **Household variables:** | |  |  |  |  |  |
| Income (000’s) | 0.018** | (0.006) | 0.002 | (0.013) | -0.020 | (0.012) |
| Calls to household | -0.003 | (0.003) | 0.002 | (0.005) | 0.001 | (0.005) |
| N  Households  Wald chi-sq (df=20)  Adjusted McFadden R-Squared  Log likelihood: Model  Log likelihood: Intercept Only | | 1301 |  |  |  |  |
| Household | | 1169 |  |  |  |  |
| *Note:* Base categories for female, dyads and region variables, are male, spousal dyad and England, respectively. The sample is smaller relative to 1384 in the main analysis sample as some respondents have a zero weight by design. Standard errors clustered at the household level in parentheses: *p<0.05; **p<0.01, ***p<0.001 | | | | | | |

| Table A9: Average marginal effects from two discrepancy models estimated separately | | | | | | | | |
| --- | --- | --- | --- | --- | --- | --- | --- | --- |
|  | Unconfirmed recipient | | | | Unconfirmed provider | | | |
| Dependent variable | (y=1 if confirmed dyad; y=0 if unconfirmed recipient dyad) | | | | (y=1 if confirmed dyad; y=0 if unconfirmed provider dyad) | | | |
|  | Basic set | | Extended set | | Basic set | | Extended set | |
|  | Marginal eff | Std err | Marginal eff | Std err | Marginal eff | Std err | Marginal eff | Std err |
| **Basic set:** | | | | | | | | |
| **Provider variables**: |  |  |  |  |  |  |  |  |
| Female | -0.04 | (0.06) | -0.01 | (0.05) | -0.09 | (0.07) | -0.07 | (0.06) |
| Age | 0.00 | (0.00) | -0.00 | (0.00) | -0.00* | (0.00) | -0.00 | (0.00) |
| **Recipient variables:** |  |  |  |  |  |  |  |  |
| Female | -0.03 | (0.06) | -0.03 | (0.05) | -0.13 | (0.07) | -0.11 | (0.06) |
| Age | 0.00 | (0.00) | -0.00 | (0.00) | 0.01*** | (0.00) | 0.00 | (0.00) |
| **Dyad variables:** |  |  |  |  |  |  |  |  |
| Adult child - parent | 0.07 | (0.09) | 0.09 | (0.08) | 0.10 | (0.08) | 0.07 | (0.07) |
| Other | -0.15 | (0.09) | -0.12* | (0.06) | -0.00 | (0.10) | 0.01 | (0.09) |
| **Household variables:** | |  |  |  |  | **variables:** |  |  |
| Household size | -0.34*** | (0.07) | -0.26*** | (0.07) | -0.77*** | (0.08) | -0.70*** | (0.07) |
| Wales | -0.03 | (0.06) | -0.05 | (0.04) | 0.06 | (0.05) | 0.01 | (0.04) |
| Scotland | 0.01 | (0.06) | 0.02 | (0.04) | 0.06 | (0.05) | 0.00 | (0.04) |
| Northern Ireland | 0.04 | (0.05) | -0.03 | (0.05) | -0.04 | (0.05) | -0.04 | (0.04) |
| **Extended set** | | | | | | | | |
| **Provider variables:** |  |  |  |  |  |  |  |  |
| Ethnic group: UK |  |  | -0.01 | (0.06) |  |  | 0.01 | (0.05) |
| Degree qualification |  |  | 0.06 | (0.03) |  |  | 0.07** | (0.03) |
| Retired |  |  | 0.08 | (0.04) |  |  | -0.03 | (0.04) |
| Health condition |  |  | -0.06* | (0.03) |  |  | 0.02 | (0.02) |
| Carer benefit |  |  | -0.07 | (0.04) |  |  | 0.04 | (0.06) |
| Others present |  |  | 0.03 | (0.03) |  |  | -0.00 | (0.02) |
| No previous interview |  |  | -0.48*** | (0.10) |  |  | -0.05 | (0.07) |
| **Recipient variables:** |  |  |  |  |  |  |  |  |
| Ethnic group: UK |  |  | -0.02 | (0.07) |  |  | 0.13* | (0.05) |
| Degree qualification |  |  | -0.03 | (0.04) |  |  | -0.03 | (0.03) |
| Number of ADLs |  |  | 0.05 | (0.03) |  |  | 0.02** | (0.01) |
| Number of IADLs |  |  | 0.10*** | (0.01) |  |  | 0.04*** | (0.01) |
| Memory difficulty |  |  | 0.00 | (0.03) |  |  | 0.12*** | (0.03) |
| Sight difficulty |  |  | -0.04 | (0.04) |  |  | 0.08* | (0.04) |
| Recipient benefit |  |  | -0.00 | (0.04) |  |  | 0.04 | (0.03) |
| Others present |  |  | 0.01 | (0.03) |  |  | 0.03 | (0.02) |
| No previous interview |  |  | 0.95*** | (0.16) |  |  | 0.05 | (0.08) |
| **Dyad variables:** |  |  |  |  |  |  |  |  |
| Int date difference |  |  | -0.06 | (0.04) |  |  | -0.05 | (0.05) |
| **Household variables:** |  |  |  |  |  |  |  |  |
| Income (000’s) |  |  | -0.02 | (0.01) |  |  | -0.01 | (0.01) |
| Calls to house |  |  | -0.00 | (0.01) |  |  | 0.00 | (0.00) |
| **Provider hours variables:** | |  |  |  |  |  |  |  |
| 5-9 hours |  |  | 0.01 | (0.03) |  |  |  |  |
| 10-19 hours |  |  | -0.02 | (0.04) |  |  |  |  |
| 20-34 hours |  |  | -0.01 | (0.05) |  |  |  |  |
| 35-49 hours |  |  | -0.05 | (0.08) |  |  |  |  |
| 50-99 hours |  |  | -0.01 | (0.11) |  |  |  |  |
| 100+ hours |  |  | -0.09 | (0.05) |  |  |  |  |
| Varies under 20 hours |  |  | -0.03 | (0.07) |  |  |  |  |
| Varies over 20 hours |  |  | 0.03 | (0.05) |  |  |  |  |
| Other hours |  |  | -0.16 | (0.09) |  |  |  |  |
| **Recipient hours variables:** | |  |  |  |  |  |  |  |
| Less than one hour |  |  |  |  |  |  | 0.06 | (0.04) |
| 1-4 hours  5-9 hours |  |  |  |  |  |  | 0.17*** | (0.04) |
| 5-9 hours |  |  |  |  |  |  | 0.34*** | (0.05) |
| 10-19 hours |  |  |  |  |  |  | 0.37*** | (0.06) |
| 20-34 hours |  |  |  |  |  |  | 0.38*** | (0.06) |
| 35-49 hours |  |  |  |  |  |  | 0.47*** | (0.08) |
| 50-99 hours |  |  |  |  |  |  | 0.48*** | (0.10) |
| 100 + hours |  |  |  |  |  |  | 0.51*** | (0.07) |
| N | 528 |  | 528 |  | 1200 |  | 1200 |  |
| Households | 499 |  | 499 |  | 1030 |  | 1030 |  |
| AIC | 2722.30 |  | 2659.47 |  | 8133.52 |  | 7656.89 |  |
| BIC | 2773.53 |  | 2791.82 |  | 8194.60 |  | 7814.68 |  |
| *Note:* Base categories for female, dyads and region variables, are male, spousal dyad and England, respectively Standard errors clustered at the household level in parentheses: *p<0.05; **p<0.01; ***p<0.001 | | | | | | | | |

Table A10: Interval regression specification with the hours of informal care provision and receipt as the dependent variable

|  | Provider hours | | | | Recipient hours | | | |
| --- | --- | --- | --- | --- | --- | --- | --- | --- |
|  | Basic set | | Extended set | | Basic set | | Extended set | |
| **Basic set** | | | | | | | | |
| **Provider variables**: |  |  |  |  |  |  |  |  |
| Female | 13.09* | (5.97) | 14.10** |  | 5.47* | (2.49) | 5.79** | (2.20) |
| Age | -0.09 | (0.27) | 0.10 |  | 0.04 | (0.12) | 0.24* | (0.11) |
| **Recipient variables:** |  |  |  |  |  |  |  |  |
| Female | 9.01 | (5.98) | 9.03 |  | 3.99 | (2.57) | 3.63 | (2.26) |
| Age | 0.45 | (0.39) | -0.27 |  | 0.28 | (0.16) | -0.30* | (0.14) |
| **Dyad variables:** |  |  |  |  |  |  |  |  |
| Adult child - parent | -25.76** | (9.71) | -25.48** |  | 1.00 | (4.20) | -0.11 | (3.51) |
| Other | -26.62** | (9.08) | -21.47** |  | -1.50 | (5.61) | -0.68 | (4.30) |
| **Household variables:** | |  |  |  |  |  |  |  |
| Household size | 3.59 | (5.42) | 8.21 |  | -0.08 | (0.90) | -2.05* | (0.91) |
| Wales | -0.48 | (6.27) | 6.46 |  | 1.04 | (2.82) | 2.11 | (2.51) |
| Scotland | 15.37 | (8.90) | 13.39 |  | 0.92 | (3.14) | -0.18 | (2.49) |
| Northern Ireland | 12.66 | (8.66) | 8.85 |  | -1.44 | (2.97) | -2.88 | (2.57) |
| **Extended set** | | | | | | | | |
| **Provider variables:** |  |  |  |  |  |  |  |  |
| Ethnic group: UK |  |  | -6.65 | (9.11) |  |  | 1.14 | (3.02) |
| Degree qualification |  |  | 4.45 | (4.99) |  |  | 1.86 | (1.76) |
| Retired |  |  | -9.15 | (6.27) |  |  | -3.45 | (2.25) |
| Health condition |  |  | 4.18 | (3.88) |  |  | -0.24 | (1.35) |
| Carer benefit |  |  | 14.84* | (6.92) |  |  | 11.10* | (4.33) |
| Others present |  |  | 2.55 | (4.46) |  |  | 1.70 | (1.41) |
| No prev int |  |  | -28.61* | (14.51) |  |  | -2.88 | (3.29) |
| **Recipient variables:** |  |  |  |  |  |  |  |  |
| Ethnic group: UK |  |  | 6.26 | (9.03) |  |  | 0.99 | (3.19) |
| Degree qualification |  |  | -2.81 | (6.17) |  |  | -0.53 | (1.92) |
| Number of ADLs |  |  | 3.92*** | (1.04) |  |  | 4.01*** | (0.62) |
| Number of IADLs |  |  | 4.91** | (1.60) |  |  | 6.87*** | (0.73) |
| Memory difficulty |  |  | 7.77 | (4.90) |  |  | 3.82 | (2.50) |
| Sight difficulty |  |  | -3.33 | (4.96) |  |  | -1.86 | (2.71) |
| Recipient benefit |  |  | 16.78** | (5.29) |  |  | 5.90* | (2.72) |
| Others present |  |  | 2.59 | (4.65) |  |  | 2.43 | (1.44) |
| No prev int |  |  | 24.88 | (18.63) |  |  | -5.46 | (5.16) |
| **Dyad variables:** |  |  |  |  |  |  |  |  |
| Int date difference |  |  | 3.28 | (6.81) |  |  | 2.64 | (2.70) |
| **Household variables:** |  |  |  |  |  |  |  |  |
| Income (000’s) |  |  | 0.30 | (2.77) |  |  | -1.44* | (0.61) |
| Calls to house |  |  | 0.74 | (0.82) |  |  | 0.22 | (0.28) |
| _cons | -5.41 | (24.50) | 0.13 | (26.05) | -13.57 | (9.73) | -0.33 | (8.76) |
| Insigma cons | 3.76*** | (0.04) | 3.65*** | (0.04) | 3.31*** | (0.05) | 3.09*** | (0.04) |
| N | 528 |  | 528 |  | 1200 |  | 1200 |  |
| Households | 499 |  | 499 |  | 1030 |  | 1030 |  |
| AIC | 2722.30 |  | 2659.47 |  | 8133.52 |  | 7656.89 |  |
| BIC | 2773.53 |  | 2791.82 |  | 8194.60 |  | 7814.68 |  |
| *Note:* Base categories for female, dyads and region variables, are male, spousal dyad and England, respectively Standard errors clustered at the household level in parentheses: *p<0.05; **p<0.01; ***p<0.001 | | | | | | | | |

Table A11: Predictors of a dyad being in the top and bottom 5^th^ percentile difference of reported hours between providers and recipients estimated via OLS using a linear probability model.

|  | Coefficient | Std err | Coefficient | Std err | | |  |
| --- | --- | --- | --- | --- | --- | --- | --- |
| **Basic set:** | | |  |  | | |  |
| **Provider variables:** |  |  |  |  | | |  |
| Female | -0.10 | (0.07) |  |  | | |  |
| Age | 0.00 | (0.00) |  |  | | |  |
| **Recipient variables:** |  |  |  |  | | |  |
| Female | -0.12 | (0.07) |  |  | | |  |
| Age | -0.00 | (0.00) |  |  | | |  |
| **Dyad variables:** |  |  |  |  | | |  |
| Parent | 0.05 | (0.08) |  |  | | |  |
| Other | 0.00 | (0.10) |  |  | | |  |
| **Household variables:** |  |  |  |  | | |  |
| Household size | 0.35 | (0.32) |  |  | | |  |
| Wales | 0.03 | (0.06) |  |  | | |  |
| Scotland | 0.06 | (0.07) |  |  | | |  |
| Northern Ireland | -0.10*** | (0.03) |  |  | | |  |
| **Extended set:** | | |  |  | | |  |
| **Provider variables:** | |  |  |  | | |  |
| Degree qualification | -0.02 | (0.04) |  |  | | |  |
| Retired | -0.03 | (0.05) |  |  | | |  |
| Health condition | 0.05 | (0.03) |  |  | | |  |
| Carer benefit | 0.05 | (0.06) |  |  | | |  |
| Others present | -0.07 | (0.04) |  |  | | |  |
| No previous interview | 0.11 | (0.09) |  |  | | |  |
| **Recipient variables:** | |  |  |  | | |  |
| Degree qualification | 0.03 | (0.06) |  |  | | |  |
| Number of ADLs | -0.01 | (0.01) |  |  | | |  |
| *Help managing stairs* |  |  | -0.01 | (0.04) | | |  |
| *Help getting around house* |  |  | -0.01 | (0.09) | | |  |
| *Help getting in/out bed* |  |  | 0.10 | (0.06) | | |  |
| *Help cutting toenails* |  |  | -0.02 | (0.04) | | |  |
| *Help with bathing/showering* |  |  | -0.03 | (0.04) | | |  |
| *Help using the toilet* |  |  | -0.01 | (0.09) | | |  |
| *Help eating* |  |  | 0.02 | (0.07) | | |  |
| *Help washing* |  |  | -0.03 | (0.10) | | |  |
| *Help getting dressed* |  |  | -0.03 | (0.04) | | |  |
| *Help taking right medicine* |  |  | -0.03 | (0.05) | | |  |
| Number of IADLs | 0.03* | (0.01) |  |  | | |  |
| *Help walking down the road* |  |  | -0.03 | (0.04) | | |  |
| *Help shopping* |  |  | 0.12*** | (0.03) | | |  |
| *Help with housework* |  |  | 0.05 | (0.03) | | |  |
| *Help with paperwork* |  |  | -0.00 | (0.04) | | |  |
| Memory difficulty | -0.00 | (0.04) |  |  | | |  |
| Sight difficulty | 0.11* | (0.05) |  |  | | |  |
| Recipient benefit | -0.02 | (0.04) |  |  | | |  |
| Others present | 0.02 | (0.04) |  |  | | |  |
| No previous interview | -0.26* | (0.11) |  |  | | |  |
| **Dyad variables:** |  |  |  |  | | |  |
| Interview date difference | 0.13 | (0.09) |  |  | | |  |
| **Household variables:** | |  |  |  | | |  |
| Income (000’s) | 0.01 | (0.02) |  |  | | |  |
| Calls to house | -0.00 | (0.01) |  |  | | |  |
| N | 404 |  |  |  | | |  |
| Households | 394 |  |  |  | | |  |
| *Note:* The difference is predicted provider hours minus predicted recipient hours. Base categories for female, dyads and region variables, are male, spousal dyad and England, respectively. Standard errors are clustered at the household level in parentheses: *p<0.05; **p<0.01, ***p<0.001 | | | | |  |  |  |
